# Phytochemical Screening and Potential Antibacterial Activity of Defatted and Nondefatted Methanolic Extracts of Xao Tam Phan (*Paramignya trimera* (Oliv.) Guillaum) Peels against Multidrug-Resistant Bacteria

**DOI:** 10.1155/2021/4233615

**Published:** 2021-08-31

**Authors:** Van-Anh Le Thi, Ngoc-Lien Nguyen, Quang-Huy Nguyen, Quyen Van Dong, Thi-Yen Do, Kieu-Oanh Nguyen T.

**Affiliations:** ^1^Department of Life Sciences, University of Science and Technology of Hanoi, Vietnam Academy of Science and Technology, 18 Hoang Quoc Viet, Cau Giay, Hanoi, Vietnam; ^2^LMI-DRISA, University of Science and Technology of Hanoi, Vietnam Academy of Science and Technology, 18 Hoang Quoc Viet, Cau Giay, Hanoi, Vietnam; ^3^Institute of Biotechnology, Vietnam Academy of Science and Technology, 18 Hoang Quoc Viet, Cau Giay, Hanoi, Vietnam

## Abstract

Xao tam phan (*Paramignya trimera* (Oliv.) Guillaum) is a traditional herbal medicine in Vietnam. Previous investigations reported mainly compounds and bioactivities of roots, stems, and leaves while there is limited information about those of fruits. This study aims to reveal the difference in the chemical profile of defatted peel (DP) and nondefatted peel (NDP) methanolic extracts of *P. trimera* using colorimetric reactions and liquid chromatography coupled with high-resolution mass spectrometry (LC-HRMS) analysis. We also showed the potential antibacterial activity of two extracts against clinically isolated bacteria strains including *P. aeruginosa*, *Salmonella* sp., and *S. aureus* with the MIC values < 100 *μ*g/mL. This preliminary result proves the traditional usage of this herbal medicine and can be helpful for further investigation on the isolation and identification of the new compounds in *P. trimera* peels.

## 1. Introduction

The threat posed by antibiotic resistance became particularly critical in recent years because multiple and extended resistant bacteria, called “superbugs,” have become more prevalent, leading to more common hard-to-treat infections all over the world [1, 2]. This situation is a global problem but particularly pressing in developing countries where the infectious disease burden is high. Among these countries, Vietnam already experiences high antibiotic resistance levels, and an alarming increase of superbugs, resistant to powerful antibiotics, has been reported [3, 4]. Seriously, the COVID-19 situation has also aggravated antibiotic resistance. Statistics show that 50% of COVID-19 mortalities suffered from secondary infections caused by multidrug-resistant microorganisms (bacterial or fungal) or coinfections [5]. There is thus an urgent need to develop new antibacterial agents, especially against Gram-negative strains. While the investigation based on soil actinomycetes, the primary source of antibiotics, is useless because of the high rediscovery rate of known compounds [6], raising a novel candidate to conquer multidrug-resistant bacteria was thus crucial. Plant resource has long been used as herbal medicine to treat a vast array of inflammatory and bacterial infections [7]. Eventhough plants can produce a variety of secondary metabolites that are active to defend insects, microorganisms, herbivores, plant pathogens, and against human pathogens, the current study on the antimicrobial potential of plants is merely the tip of the iceberg.

*Paramignya trimera* (Oliv.) Guillaum, locally named “Xao tam phan,” belongs to the *Paramignya* genus of the Rutaceae family, mainly in the south of Vietnam [8]. This native plant is a traditional remedy used for liver protection and infectious disease treatment. Recently, various potential bioactivities, i.e., antioxidant, antibacterial, and anticancer activities and hepatoprotective property, were reported from the plant's roots, leaves, and stems. Many compounds that belong to coumarins, acridone alkaloids, phenols, flavonoids, and chromenes classes have been isolated from these tissues (Table 1S, Supplementary Materials) [9–19]. Furthermore, being a perennial woody plant, the exploitation of this plant from other tissues, i.e., fruits, instead of roots, leaves, or stems, may provide a chance to conserve this valuable medicinal plant. However, the research on the bioactivity and chemical composition of *P. trimera* fruits has been limited. This study thus aims to phytochemically dereplicate the defatted peel (DP) and nondefatted peel (NDP) methanolic extracts of *P. trimera* and to determine the activity against multidrug-resistant bacterial strains clinically isolated from patients in Vietnam.

## 2. Materials and Methods

### 2.1. Plant Materials

The *Paramignya trimera* fresh fruits were collected from the Ninh Hoa district, Khanh Hoa province, Vietnam, in August 2020. The samples were identified by Khanh Hoa Traditional Medicine Association, Khanh Hoa province, Vietnam. The voucher of specimens was deposited in the Department of Life Sciences, University of Science and Technology of Hanoi, Vietnam Academy of Science and Technology.

The whole fruits were cleaned in tap water, rinsed by distilled water to remove dust, and then separated into peel and seed. These tissues were ground into smaller pieces and stored at −80°C until used for further analysis.

### 2.2. Microbial Materials

Bacterial strains including *Acinetobacter baumannii*, *Pseudomonas aeruginosa, Escherichia coli, Staphylococcus aureus*, and *Salmonella sp.* were kindly provided from LMI DRISA (Laboratoire Mixte International on Drug Resistant in South Asia, University of Science and Technology of Hanoi). These bacteria originally were isolated clinically from different hospitals in Hanoi, Vietnam, and maintained on the nutrient solution at a −80°C deep freezer until experiments. The susceptibility test result of these strains against different antibiotics is provided in Table 2S (Supplementary Materials).

### 2.3. Sample Extraction

The fresh peels including the defatted peel (DP, defatted by *n*-hexane) and nondefatted peel (NDP) of *P. trimera* (20 g) were immersed in 100 mL of MeOH at room temperature for 24 hours. This process was repeated three times to extract the bioactive compounds maximally. The combined extracts were then filtered using the 0.45 *μ*m cellulose acetate membrane to remove all the particulars and concentrated in the Buchi Rotavapor (Flawil, Switzerland) to obtain crude extracts. These extracts were dried under nitrogen gas blowing at room temperature to a constant weight.

### 2.4. Phytochemical Screening

Phytochemical screening of the abovementioned extracts was performed using the conventional protocol and reagents. Alkaloids were detected using Bouchardat/Mayer/Dragendorff tests. Indeed, to a small amount of each sample was added gently several drops of each reagent, Bouchardat, Mayer, and Dragendorff, in a test tube; the formation of brown/white/orange precipitate, respectively, confirms the presence of alkaloids. Flavonoids were identified by adding 2 mL of 10% NaOH into the extracts; a yellow colour formation indicates the presence of flavonoids. This solution becomes colourless when adding few drops of 10% v/v HCl solution. Glycosides were detected by mixing 5 mL of each extract and 25 mL of 10% v/v H_2_SO_4_ solution. This mixture was heated to its boiling point for 15 minutes and then cooled and neutralized with 10% w/v NaOH solution. 5 mL of Fehling solution was added to it, and the red brick precipitate indicated the presence of glycosides. For terpenoids, 2 mL of dichloromethane was added to 5 mL of each extract, followed by carefully adding concentrated H_2_SO_4_. The formation of a reddish-brown colour layer confirms the presence of terpenoids. The FeCl_3_ test was used to identify tannins. A small amount of each extract is diluted and filtered, and then, few drops of 10% w/v solution of FeCl_3_ were added. The appearance of blue or green colour suggests the presence of tannins in the extract. For coumarins, 3 mL of 10% NaOH was added to an aqueous plant extract, and yellow colour was observed in positive results. Saponins identified by shaking the mixture of 2 mL of alcohol diluted with water is added to 2 mL of the plant extract and shacked well for 15 minutes in a graduated cylinder. The formation of a layer of foam (approximately 1 cm) indicates the presence of saponins. Steroids were detected by treating each extract with few drops of concentrated H_2_SO_4_ in dichloromethane; the appearance of red colour in the chloroform layer indicated the presence of steroids. Anthraquinones were detected by the Borntrager test, in which 1 mL of the ethyl-acetate fraction of each extract was added to 10 mL of 10% v/v NH_4_OH solution. The formation of pink/red/violet colour indicates the presence of anthraquinones.

### 2.5. Determination of Antibacterial Activity of the Extracts

The inhibition percentage against bacterial strains was evaluated by the microbroth dilution assay by Sultanbawa et al. with some modifications [20]. First, the bacteria stock solution was taken out from the deep freezer and placed at room temperature. The 30 *μ*L bacterial supernatant was transferred into flash bottles containing 50 mL tryptic soy broth (TSB) and incubated under shaking condition (150 rpm) at 37°C overnight. After 24 h, the density of bacterial suspension was adjusted to equal the value of the 0.5 McFarland standards corresponding to the final concentration of 10^6^ CFU/mL (colony-forming unit).

The crude extracts (DP and NDP) were first dissolved in dimethyl sulfoxide (DMSO) at 20480 *μ*g/mL and then diluted in the TSB medium at 1024 *μ*g/mL. Wells were filled with 100 *μ*L of each solution and 100 *μ*L bacterial suspension. Ciprofloxacin at a 30 *μ*g/mL concentration was used as a positive control, while 2.5% DMSO was used as a negative control. Each plate well was normalized by the subtracted blank samples. Subsequently, the microplates were incubated at 37°C for 24 hours, and the samples' absorbance was measured at 600 nm using an iMark microplate reader (BioRad, California, USA). The inhibition percentage was calculated using the following formula: %inhibition = (Ab_DMSO−Ab_sample)/Ab_DMSO × 100%, where Ab_ DMSO and Ab_sample are the absorbance values of the negative control and the sample, respectively.

The inhibition percentage of a series of dilutions ranging from 512 to 16 *μ*g/mL was evaluated. The MIC value was defined as the concentration of extract exhibiting 70–80% bacterial inhibition after 24 hours of incubation at 37°C. The MBC value was identified as the lowest concentration of extracts reducing the initial bacterial inoculum viability by more than 99.9% after 24 hours [21]. These values can be determined from the microbroth dilution of MIC tests. In plates containing the TSB medium, 10 *μ*L of culture taken from each well in the MIC range was inoculated. These plates were then incubated at 37°C for 24 h. The lowest concentration of which each extract did not show any bacterial growth was regarded as their MBC values.

### 2.6. LC-HRMS Analysis

The extracts were dissolved in MeOH (HPLC grade) to 100 mg/mL solution, filtered by 0.22 *μ*m cellulose acetate membrane, and then transferred into the 2-mL vials for injection into the LC-HRMS system. The HPLC-DAD-ESI/QTOF-MS/MS analysis was performed on an ExionLC system coupled to an ExionLC DAD detector and equipped with a high-resolution X500 QTOF mass spectrometer (Sciex, USA). The SCIEX OS 1.0 software from Sciex (Sciex, USA) contains instrument control, data acquisition, data processing, and reporting functionality, all in one package. Chromatographic separation was achieved on a Kinetex XB-C18 100 Å column (100 mm × 2.1 mm, 1.7 *μ*m) (Phenomenex). A binary mobile solvent was used: solvent *A* (H_2_O) and solvent *B* (MeOH). The mobile phase was pumped at a flow rate of 0.3 mL/min with a gradient elution profile that began at 20% *B* at 5 min, then linearly ramped to 80% *B* within 5 min, ramped to 100% *B* in 5 min, and held at 50% *B* for 5 min; then, the column was reequilibrated at 25% *B* for 5 min before the next injection. The autosampler tray temperature was set to 15°C, while the column temperature was 15°C. The injection volume was 10 *μ*L.

The signals were detected at a wavelength *λ* 254 nm. The QTOF HRMS was equipped with a TurbolonSpray ion source, and the ESI negative mode was applied, scanning spectra from m/z 100 to 3000. The spray voltage and ion source temperature were set to 4500 V and 450°C, respectively. The ion source gas 1, ion source gas 2, curtain gas, and CAD gas were set to 50, 50, 25, and 7 psi, respectively. Metabolites annotation was based on UV, HRMS, MSMS spectra, and relative RTs.

### 2.7. Statistical Analysis

All experiments were performed in triplicate. The obtained data were analyzed using Microsoft Office Excel 2016 for statistical analysis. The independent Student's *t*-test and the one-way ANOVA test were used to assess the differences among different concentrations and between the test and negative control groups.

## 3. Results and Discussion

### 3.1. Phytochemical Screening

Preliminary phytochemical screening showed several groups of metabolites such as alkaloids, flavonoids, glycosides, terpenoids, coumarins, and steroids in the methanolic extracts of the nondefatted and defatted peels of *P. trimera*. The result of the phytochemical test is provided in Table 1.

Two extracts were rich in flavonoids and glycosides. These tests also showed relatively that alkaloids, flavonoids, and glycosides were found with higher concentrations in DP than NDP. Terpenoids and steroids, known as nonpolar compounds, dominate logically in nondefatted portion NDP rather than in DP. This phytochemical screening result was consistent with previous studies showing the profiles of roots and stems of *P. trimera* [9–19].

### 3.2. Antibacterial Activity of *P. trimera* Extracts

In 2017, the WHO published its first list of antibiotic-resistant “priority pathogens,” a catalogue of 12 families of bacteria that pose the greatest threat to human health drawn up to guide and promote research and development of new antibiotics. According to the priority list of antibiotic-resistant bacteria, we focused on the five following most problematic bacteria in Vietnam: *A. baumannii*, *P. aeruginosa*, *S. aureus*, *E. coli*, and *Salmonella* sp. Indeed, eight clinical strains of the mentioned five species were used as models to test the antibacterial activity of the extracts. At the first screening step, the inhibition percentage of NDP and DP at the concentration of 512 mg/mL was nearly 100% against six multidrug-resistant strains, including *P. aeruginosa* PA1 and PA2, *S. aureus* SA1 and SA2, and *Salmonella* sp. SS1 and SS2 (Table 3S, Supplementary Materials). In particular, the DP represented a more substantial inhibitory capacity than NDP extracts, indicating that removing nonpolar compounds helps increase the activity.

These extracts were then subjected to determine the minimum inhibitory concentration (MIC) and minimum bactericidal concentration (MBC) values on six bacterial strains. Overall, all mentioned extracts can be considered bactericidal agents against six clinically dangerous bacteria in this study because their MBC values were no more than four times their MIC values. As given in Table 2, these extracts presented the MIC values globally ranging from 16 to 320 mg/mL. The DP exhibited the highest antibacterial activity on all six strains with the MIC value < 80 mg/mL, especially against four strains, i.e., *P. aeruginosa* PA1, *S. aureus* SA2, and *Salmonella* sp. SS 1 and SS2 with the MIC < 32 mg/mL. At the same time, eventhough NDP did not show the potential activity on PA2 and SA1, this extract inhibited effectively four remaining strains with the MIC < 32 mg/mL. Indeed, the MIC variation followed a similar rule, horizontally and vertically, in Table 2, suggesting that the mechanism of action of these extracts seems to be the same on all six strains. The antibacterial activity was thus contributed by the same compounds pattern. Regarding the chemical profile of the extracts, we hypothesize that alkaloids may not be responsible for the bacterial inhibition activity, and this property can be due to flavonoids and coumarins.

*P. aeruginosa* is recognized as a common Gram-negative bacterium, an opportunistic pathogen associated with a range of nosocomial infections. In this study, the tested strains were isolated clinically from patients, with remarkable resistant mechanisms to many antibiotic classes (Table 2S, Supplementary Materials). Regarding the difference in the susceptibility tests, *P. aeruginosa* PA1 was resistant to all tested antibiotics except aztreonam. In contrast, PA2 was less resistant to quinolones (norfloxacin, ciprofloxacin, and levofloxacin). The inhibition activity of the extracts against PA2 was higher than against PA1 and allowed us to hypothesize that the mechanism of action of these samples may be similar to that of quinolones. On the other hand, *Salmonella* sp. SS1 was sensitive to nalidixic acid, and SS2 was resistant to that antibiotic (Table 2S, Supplementary Materials). The MIC and MBC values of the extracts against these strains were relatively equal, suggesting that the activity may be controlled by different mechanisms with that of nalidixic acid. In the context that the effort to find out antibacterial agents against Gram-negative strains does not bring much hope in recent years, this result can be open to further more in-depth research on this studied plant tissue. For *S. aureus*, a Gram-positive species, the antibacterial activity of the extracts against SA2 was stronger than against SA1. The inhibition against *S. aureus* of the essential oil extracted from *P. trimera* leaves has been reported in the previous study [22]; however, it is the first study that indicates the potential antibacterial activity of peels of this plant. This is worth waiting because both SA1 and SA2 were dangerous multidrug-resistant bacteria (Table 2S, Supplementary Materials).

### 3.3. LC-HRMS Analysis

**Figure 1:**
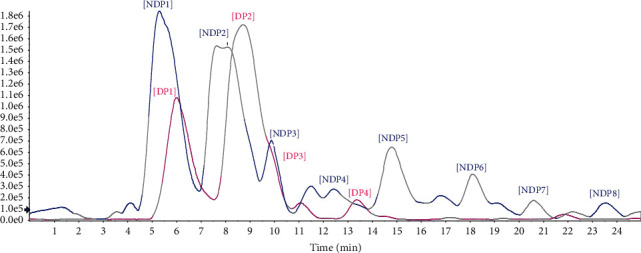
Total ion chromatograms of NDP (in blue) and DP samples (in pink) analyzed by HPLC-ESI-QTOF mass spectrometry.

To go deeply into the phytochemical composition of these extracts above, we dereplicated known metabolites using LC-HRMS analysis. The exact mass of detected components was compared to those of reference compounds shown in previous studies [9–19] (Table 1S, Supplementary Materials). The QTOF MS-based chromatograms on nondefatted and defatted peels clearly showed similar and different segments between the two samples (Figure 1). Eventhough there is a slight shift in the peaks' retention time, two chromatograms demonstrated the same pattern in the first elution segment. The chromatogram of DP shows fewer peaks than that of the NDP sample due to the removal of hydrophobic compounds. Indeed, the peak eluted at 5.4 min in NDP and 5.8 min in the DP sample showed the base peak of molecular ion [M-H-] at m/z 191.0555, corresponding to the formula C_7_H_12_O_6_. We annotated this peak as quinic acid based on the similarities in MS/MS fragmentation between the obtained peak and reference (Table 3). The peak NDP3 and DP3 showed the base peak at m/z 283.2624, which corresponds to C_18_H_36_O_2_. The peaks NDP6, NDP7, and NDP8 showed the base peaks at m/z 367.3567, 381.3724, and 395.3879, respectively, corresponding to C_24_H_48_O_2_, C_25_H_50_O_2_, and C_26_H_52_O_2_. These formulas contain only two oxygens, one double bond, eluted at the end of chromatograms separated by reverse phase column and were detected only in the NDP samples suggesting that they were nonpolar metabolites. We annotated them as fatty acids: NDP3/DP3 (stearic acid), NDP6 (tetracosanoic acid), NDP7 (pentacosanoic acid), and NDP8 (hexacosanoic acid). The inhibitory effect of DP extract was more potent than NDP extract, suggesting that fatty acids do not contribute to the antibacterial activity. It was consistent with the hypothesis that antibacterial potential may be responsible by flavonoids and coumarins in *P. trimera* peel. Noticeably, the LC-HRMS analysis showed that detected peaks in both defatted and nondefatted peel extracts did not match to any of known metabolites previously isolated from other *P. trimera* tissues (Table 1S, Supplementary Materials). They have probably been never investigated in the peel of this medicinal plant; therefore, further studies should be performed to identify these compounds as well as its bioactivities.

## 4. Conclusions

For the first time, the potential inhibitory effect of the peels extracts of *P. trimera* against six MDR bacterial strains isolated clinically from hospitals in Vietnam, especially Gram-negative *P. aeruginosa* strains, was reported. Removal of the fatty portion of the peels could enhance the effectiveness against multidrug-resistant bacteria suggesting that the flavonoids and coumarins dominating in the DP may be responsible for the activity. This study contributes to the scientific validity of *P. trimera* being used traditionally as a medicine and provides the guide for further investigation of new compounds in *P. trimera* to develop new treatment options against multidrug-resistant bacteria.

## Figures and Tables

**Table 1:** Phytochemical analysis of the nondefatted peel (NDP) and defatted peel (DP) of *P. trimera*.

Test	Observation	NDP	DP
Alkaloid			
Bouchardat	Formation of brown precipitate	—	—
Mayer	Formation of yellow-white precipitate	—	+
Dragendorff	Formation of orange precipitate	—	+
Flavonoids			
Alkaline reagent	Appearance of yellow colour, become colourless when adding diluted HCl solution	++	+++
Glycosides			
Salkowski	Appearance of reddish colour	++	+++
Terpenoids	Formation of grey colour	++	+
Tannins	Formation of blue or green colour	—	—
Coumarins	Formation of yellow colour	++	++
Saponins	Formation of foam	—	—
Steroids	Appearance of red colour	++	+
Anthraquinones	Appearance of pink/red/violet solution	—	—

**Table 2:** The minimum inhibitory concentration (MIC) (*μ*g/mL) and the minimum bactericidal concentration (MBC) (*μ*g/mL) of the nondefatted peel (NDP) and defatted peel (DP) methanolic extracts of *P. trimera* against six clinically isolated bacterial strains using the broth dilution assay.

	Sample	*P. aeruginosa*		*S. aureus*		*Salmonella* sp.	
		PA2	PA1	SA1	SA2	SS2	SS1
MIC	NDP	320	24	—	32	32	32
	DP	64	16	80	16	32	32

MBC	NDP	512	64	—	64	128	128
	DP	256	64	128	32	128	64

**Table 3:** Metabolites annotation of the nondefatted peel (NDP) and defatted peel (DP) extracts of *P. trimera*.

Peak	Retention time	[M-H-] (m/z)	Formula	MS/MS fragmentation	Annotation
NDP2; DP2	8.0; 8.6	661.3898	C_43_H_52_O_3_	No fragmentation	
NDP3; DP3	10.1; 11.1	283.2624	C_18_H_36_O_2_	265.2512	Stearic acid
NDP4	12.3	399.3469	C_24_H_48_O_4_	353.3418; 323.3310; 221.1022	Dihydroxytetracosanoic acid
NDP5	14.8	383.3513	C_24_H_48_O_3_	337.3459	Hydroxytetracosanoic acid
NDP6	18.2	367.3567	C_24_H_48_O_2_	No fragmentation	Tetracosanoic acid
NDP7	20.7	381.3724	C_25_H_50_O_2_	335.2577; 245.0265	Pentacosanoic acid
NDP8	23.5	395.3879	C_26_H_52_O_2_	No fragmentation	Hexacosanoic acid
DP4	13.4	758.5365	nd		

## Data Availability

The data used to support the findings of this study are included in the Supplementary Material file.

## Supplementary Materials

Supplementary Materials The supplementary materials include 3 tables.
