# Supplementary material for: Phytochemical Screening and Potential Antibacterial Activity of Defatted and Nondefatted Methanolic Extracts of Xao Tam Phan (Paramignya trimera (Oliv.) Guillaum) Peels against Multidrug-Resistant Bacteria
Source: Scientifica (Cairo). 2021 Aug 31;2021:4233615. doi: 10.1155/2021/4233615 (PMC8424233; doi:10.1155/2021/4233615)
Supplement: Supplementary Materials — The supplementary materials include 3 tables. [file 4233615.f1.pdf]

Title and Authorship Information

Manuscript title:

Phytochemical screening and potential antibacterial activity of defatted and non-defatted methanolic extracts of Xao tam phan (*Paramignya trimera* (Oliv.) Guillaum) peels against multidrug-resistant bacteria.

Full author names, institutional mailing addresses and email addresses
Van-Anh Le Thi
Department of Life Sciences, University of Science and Technology of Hanoi, Vietnam Academy of Science and Technology, 18-Hoang Quoc Viet, Cau Giay, Hanoi, Vietnam
le-thi-van.anh@usth.edu.vn

Ngoc-Lien Nguyen
Department of Life Sciences, University of Science and Technology of Hanoi, Vietnam Academy of Science and Technology, 18-Hoang Quoc Viet, Cau Giay, Hanoi, Vietnam
rubynguyenlien@gmail.com

Quang-Huy Nguyen
Department of Life Sciences, University of Science and Technology of Hanoi, Vietnam Academy of Science and Technology, 18-Hoang Quoc Viet, Cau Giay, Hanoi, Vietnam
nguyen-quang.huy@usth.edu.vn

Van-Quyen Dong
Department of Life Sciences, University of Science and Technology of Hanoi (USTH), Vietnam Academy of Science and Technology (VAST). 18 Hoang Quoc Viet, Cau Giay, Hanoi, Vietnam
Institue of Biotechnology, Vietnam Academy of Science and Technology (VAST). 18 Hoang Quoc Viet, Cau Giay, Hanoi, Vietnam
dvquyen@gmail.com

Thi-Yen Do
Department of Life Sciences, University of Science and Technology of Hanoi, Vietnam Academy of Science and Technology, 18-Hoang Quoc Viet, Cau Giay, Hanoi, Vietnam
yen877398@gmail.com

Kieu-Oanh Nguyen T.
Department of Life Sciences, University of Science and Technology of Hanoi, Vietnam Academy of Science and Technology, 18-Hoang Quoc Viet, Cau Giay, Hanoi, Vietnam
nguyen-thi-kieu.oanh@usth.edu.vn

Correspondence should be addressed to:

Kieu-Oanh Nguyen Thi, Department of Life Sciences, University of Science and Technology of Hanoi (USTH), Vietnam Academy of Science and Technology (VAST). 18 Hoang Quoc Viet, Cau Giay, Hanoi
E-mail: nguyen-thi-kieu.oanh@usth.edu.vn
Mobile phone: +84-9 88 40 76 36
Phone & Fax: +84-24 37 91 69 60

Supplementary materials

Table 1S: Chemical composition of different tissues of *P. trimera* in previous studies

No	Compound	Monoisotopic mass	Formula	Tissue	References
**1**	rutin	610.15338	$C_{27}H_{30}O_{16}$	root	[16]
**2**	quercetin	302.04265	$C_{15}H_{10}O_7$	root	[16]
**3**	kaempferol	286.04773	$C_{15}H_{10}O_6$	root	[16]
**4**	(±)-naringenin	272.06847	$C_{15}H_{12}O_5$	root	[16]
**5**	myricetin	318.03756	$C_{15}H_{10}O_8$	root	[16]
**6**	luteolin	286.04773	$C_{15}H_{10}O_6$	root	[16]
**7**	apigenin	270.05282	$C_{15}H_{10}O_5$	root	[16]
**8**	(-)-epigallocatechin gallate	458.08491	$C_{22}H_{18}O_{11}$	root	[16]
**9**	(+)-catechin	290.07904	$C_{15}H_{14}O_6$	root	[16]
**10**	(-)-epicatechin	290.07904	$C_{15}H_{14}O_6$	root	[16]
**11**	gallic acid	170.02152	$C_7H_6O_5$	root	[16]
**12**	p-coumaric acid	164.04734	$C_9H_8O_3$	root	[16]
**13**	chlorogenic acid	354.09508	$C_{16}H_{18}O_9$	root	[16]
**14**	caffeic acid	180.04225	$C_9H_8O_4$	root	[16]
**15**	syringic acid	198.05282	$C_9H_{10}O_5$	root	[16]
**16**	5,7-dimethoxycoumarin	206.05790	$C_{11}H_{10}O_4$	root	[16]
**17**	methyl 4-hydrobenzoate	304.09468	$C_{16}H_{16}O_6$	stem	[8]
**18**	methyl p (E)-coumarate	220.07355	$C_{12}H_{12}O_4$	stem	[8]
**19**	methyl syringate	212.06847	$C_{10}H_{12}O_5$	stem	[8]
**20**	vanillin	152.04734	$C_8H_8O_3$	stem	[8]
**21**	(E)-methyl 3-(4’-hydroxy-3’,5’-dimethoxyphenyl) acrylate	238.08412	$C_{12}H_{14}O_5$	stem	[8]
**22**	methyl ferulate	208.07355	$C_{11}H_{12}O_4$	stem	[8]
**23**	methyl 4-hydroxy-3-methoxybenzoate	182.05790	$C_9H_{10}O_4$	stem	[8]
**24**	vanillic acid	168.04225	$C_8H_8O_4$	stem	[8]
**25**	escin	1130.55090	$C_{55}H_{86}O_{24}$	root	[18]
**26**	beta-sitosterol	414.38616	$C_{29}H_{50}O$	root	[18]
**27**	b-sitosterol-beta-D-glucoside	576.43899	$C_{35}H_{60}O_6$	root	[18]
**28**	7-hydroxycoumarin	162.03169	$C_9H_6O_3$	root	[9, 19]
**29**	7-methoxycoumarin	176.04734	$C_{10}H_8O_3$	root	[9, 19]
**30**	ostruthin	298.15689	$C_{19}H_{22}O_3$	root	[9, 19]
**31**	ninhvanin (8-methoxyostruthin)	329.17528	$C_{20}H_{25}O_4$	root	[9, 19]
**32**	6-(6-Hydroxy-3,7-dimethylocta-2,7-dienyl)-7-hydroxycoumarin	314.15180	$C_{19}H_{22}O_4$	root	[19]
**33**	Paratrimerin A	780.26294	$C_{40}H_{44}O_{16}$	root	[9, 19]
**34**	Paratrimerin B	912.30520	$C_{45}H_{52}O_{20}$	root	[9, 19]
**35**	Paratrimerin C	282.07660	$C_{16}H_{12}NO_4$	root	[12]
**36**	Paratrimerin D	410.19670	$C_{24}H_{28}NO_5$	root	[12]
**37**	Paratrimerin E	332.16237	$C_{19}H_{24}O_5$	root	[12]
**38**	Paratrimerin F	296.14124	$C_{19}H_{20}O_3$	root	[12]

**39**	Paratrimerin G	238.12051	$C_{13}H_{18}O_4$	root	[10]
**40**	Paratrimerin H	240.09977	$C_{12}H_{16}O_5$	root	[10]
**41**	Scopoletin	192.04225	$C_{10}H_8O_4$	root	[12]
**42**	Xanthyletin	228.07864	$C_{14}H_{12}O_3$	root	[12]
**43**	Pandanusin A	315.15963	$C_{19}H_{23}O_4$	root	[12]
**44**	8-Geranyl-7-hydroxycoumarin	298.15689	$C_{19}H_{22}O_3$	stem	[17]
**45**	6-(7-Hydroperoxy-3,7-dimethylocta-2,5-dienyl)-7-hydroxycoumarin	331.15710	$C_{19}H_{23}O_5$	stem	[17]
**46**	6-(6’,7’-Dihydroxy-3’,7’-dimethylocta-2’-enyl)-7-hydroxycoumarin	332.16237	$C_{19}H_{24}O_5$	stem	[17]
**47**	luvangetin	258.08921	$C_{15}H_{14}O_4$	stem	[17]
**48**	Citrusinine-I	301.09502	$C_{16}H_{15}NO_5$	root	[12, 17]
**49**	Glycocitrine-III	325.13140	$C_{19}H_{19}NO_4$	root	[12, 17]
**50**	Oriciacridone E	325.13141	$C_{19}H_{19}NO_4$	root	[17]
**51**	Oriciacridone	324.12110	$C_{19}H_{18}NO_4$	root	[12, 17]
**52**	5-Hydroxynoracronycin	323.11575	$C_{19}H_{17}NO_4$	root	[12, 17]
**53**	Daedalin A	192.07864	$C_{11}H_{12}O_3$	root	[17]
**54**	6-(2-hydroxyetyl)-2,2-dimetyl-2H-1-benzopyran	204.11503	$C_{13}H_{16}O_2$	root	[9]

Table 2S: Susceptibility test of eight bacterial strains against different antibiotics. (+) the strain was resistant to the antibiotic, (–) the strain was sensitive to the antibiotic, (I) the antibiotic can inhibit the strain, nd: not determined.

**Antibiotics used**	***P. aeruginosa***		***S. aureus***		***Salmonella sp***		***A. baumanii***	***E.coli***
	PA1	PA2	SA1	SA2	SS2	SS1	AB	EC
**Amikacin 30µg**	+	+	nd	nd	nd	nd	+	-
**Ampicillin 10µg**	+	+	+	+	nd	nd	+	I
**Fosfomycin 200µg**	+	+	-	+	nd	nd	+	nd
**Gentamicin 10µg**	+	+	-	+	nd	nd	+	-
**Netilmicin 10µg**	+	+	nd	nd	nd	nd	+	nd
**Tobramycin 10µg**	+	+	-	+	nd	nd	+	-
**Chloramphenicol 30µg**	+	+	nd	nd	nd	nd	nd	nd
**Meropenem 10µg**	+	+	nd	nd	nd	nd	nd	-
**Ertapenem 10µg**	+	+	nd	nd	nd	nd	+	-
**Imipenem 10µg**	+	+	nd	nd	nd	nd	+	-
**Cephalexin 30µg**	+	+	nd	nd	nd	nd	+	-
**Cefpodoxime proxetil 10µg**	+	+	nd	nd	nd	nd	+	-
**Cefoxitin 30µg**	+	+	-	+	nd	nd	+	-
**Cefsulodin 30µg**	+	+	nd	nd	nd	nd	nd	nd
**Ceftazidime 10µg**	+	+	nd	nd	nd	nd	+	nd
**Cefepime 30µg**	+	+	nd	nd	nd	nd	+	-
**Ciprofloxacin 5µg**	+	I	nd	nd	nd	nd	+	-
**Levofloxacin 5µg**	+	I	nd	nd	nd	nd	-	-
**Ofloxacin 5µg**	+	+	-	-	nd	nd	nd	-
**Norfloxacin 10µg**	+	I	nd	nd	nd	nd	nd	nd
**Nalidixic acid 30µg**	+	+	nd	nd	+	-	+	-
**Trimethoprim + sulfamothoxazole 1.25 + 23.75µg**	+	+	-	-	nd	nd	+	nd

**Aztreonam 30µg**	-	+	nd	nd	nd	nd	nd	nd
**Temocillin 30µg**	+	+	nd	nd	nd	nd	+	-
**Ticarcillin 75µg**	+	+	nd	nd	nd	nd	+	nd
**Ticarcillin + Clavulanic acid 75 µg +10µg**	+	+	nd	nd	nd	nd	+	-
**Piperacillin 30µg + Tazobactam 6ug**	I	+	nd	nd	nd	nd	+	-
**Amoxicillin + Clavulanic acid 20µg + 10µg**	+	+	-	-	nd	nd	+	I
**Piperacillin 30µg**	+	+	nd	nd	nd	nd	+	I
**Fusidic acid (10µg)**	nd	nd	-	-	nd	nd	nd	nd
**Clindamycin (2µg)**	nd	nd	-	+	nd	nd	nd	nd
**Erythromycin (15µg)**	nd	nd	-	+	nd	nd	nd	nd
**Kanamycin (30µg)**	nd	nd	+	+	nd	nd	nd	nd
**Oxacillin (5µg)**	nd	nd	-	I	nd	nd	nd	nd
**Penicillin (1µg)**	nd	nd	+	+	nd	nd	nd	nd
**Pristinamycin (15µg)**	nd	nd	-	+	nd	nd	nd	nd
**Rifampicin (5µg)**	nd	nd	-	-	nd	nd	nd	nd
**Tetracyclin (30µg)**	nd	nd	+	-	nd	nd	+	nd
**Teicoplanin (30µg)**	nd	nd	-	-	nd	nd	nd	nd
**Vancomycin (5µg)**	nd	nd	I	-	nd	nd	nd	nd
**Cefotaxime 30µg**	nd	nd	nd	nd	nd	nd	nd	nd
**Colistin 50µg**	nd	nd	nd	nd	nd	nd	nd	nd

Table 3S. The inhibition percentage of defatted peel (DP) and non-defatted peel (NDP) of *P. trimera* at a concentration of 512 µg/mL against clinically isolated bacterial strains using broth dilution assay

**Sample**	***P. aeruginosa***		***S. aureus***		***Salmonella sp***		***A. baumannii***	***E.coli***
	PA1	PA2	SA1	SA2	SS1	SS2	AB	EC
**DP**	99.98 ± 0.18	98.81 ± 1.07	42.70 ± 3.64	99.73 ± 0.57	100 ± 0	99.23 ± 1.33	45.79 ± 0.34	10.74 ± 3.19
**NDP**	95.55 ± 2.08	98.70 ± 2.24	95.63 ± 1.14	99.67 ± 0.68	100 ± 0	100 ± 0	19.06 ± 2.13	1.05 ± 2.69
**Ab**	99.88 ± 0.19	94.44 ± 2.98	99.43 ± 0.27	98.29 ± 0.13	98.33 ± 0.76	96.86 ± 2.89	67.11 ± 4.37	91.87 ± 3.12
